# COMPARISON OF TWO INHALATIONAL TECHNIQUES FOR BRONCHODILATOR ADMINISTRATION IN CHILDREN AND ADOLESCENTS WITH ACUTE ASTHMA CRISIS: A META-ANALYSIS

**DOI:** 10.1590/1984-0462/;2018;36;3;00002

**Published:** 2018

**Authors:** Cristian Roncada, Julia Andrade, Luísa Carolina Bischoff, Paulo Márcio Pitrez

**Affiliations:** a Centro Universitário da Serra Gaúcha, Caxias do Sul, RS, Brasil.; b Pontifícia Universidade Católica do Rio Grande do Sul, Porto Alegre, RS, Brasil.

**Keywords:** Nebulizer, Metered-dose inhaler, MDI, Asthma, Child, Nebulizador, Inalador dosimetrado, MDI, Asma, Criança

## Abstract

**Objective:**

To compare the efficacy of pediatric asthma treatment by nebulizer and metered-dose inhaler with the use of a spacer (MDI-spacer) in rescue techniques for asthmatic patients assisted at pediatric emergency units.

**Data sources:**

A systematic review was conducted to identify the most relevant randomized controlled trials comparing the administration of a bronchodilator (β-2 agonist) by two inhalation techniques (nebulization and MDI-spacer) to treat asthma in children at pediatric emergency units. The following databases were searched: PubMed, Scientific Electronic Library Online (SciELO), and ScienceDirect. Two researchers independently applied the eligibility criteria, and only randomized controlled trials that compared both inhalation techniques (nebulization and MDI-spacer) for asthma treatment at pediatric emergency units were included.

**Data synthesis:**

212 articles were pre-selected, of which only nine met the eligibility criteria and were included in meta-analysis. Results show no differences between inhalation techniques for any of the four outcomes analyzed: heart rate (difference - Df: 1.99 [95% confidence interval - 95%CI -2.01-6.00]); respiratory rate (Df: 0.11 [95%CI -1.35-1.56]); O_2_ saturation (Df: -0.01 [95%CI -0.50-0.48]); and asthma score (Df: 0.06 [95%CI -0,26-0.38]).

**Conclusions:**

The findings demonstrate no differences in cardiorespiratory frequency, O_2_ saturation, and asthma scores upon administration of β-2 agonist by both inhalation techniques (nebulization and MDI-spacer) to asthmatic patients assisted at pediatric emergency units.

## RESUMO

**Objetivo:**

Comparar a eficácia no tratamento da asma pediátrica por nebulizador e inalador dosimetrado com uso de espaçador (MDI-espaçador), no emprego das técnicas de resgate de pacientes asmáticos atendidos em emergências pediátricas.

**Fontes de dados:**

Realizou-se uma revisão sistemática para identificar os principais estudos randomizados controlados que comparam a administração de broncodilatador (β-2 agonista) por meio das técnicas inalatórias nebulização e MDI-espaçador no tratamento da asma em unidades de emergência pediátrica. Foram pesquisadas as bases de dados PubMed, Scientific Electronic Library Online (SciELO) e ScienceDirect. Dois pesquisadores, de forma independente, aplicaram os critérios de elegibilidade, sendo incluídos na pesquisa apenas estudos randomizados controlados com o objetivo de comparar as técnicas inalatórias nebulização e MDI-espaçador no tratamento da asma em unidades de emergência pediátrica.

**Síntese dos dados:**

Foram pré-selecionados 212 artigos, dos quais apenas nove seguiram os critérios de elegibilidade e foram incluídos na metanálise. Os resultados apontam não existir diferenças nas técnicas inalatórias em nenhum dos quatro desfechos analisados: frequência cardíaca (diferença -Df: 1,99 [intervalo de confiança de 95% - IC95% -2,01-6,00]); frequência respiratória (Df: 0,11 [IC95% -1,35-1,56]); saturação de O_2_ (Df: -0,01 [IC95% -0,50-0,48]); e escore clínico de asma (Df: 0,06 [IC95% -0,26-0,38]).

**Conclusões:**

Os achados demonstram não haver diferenças na frequência cardiorrespiratória, na saturação de O_2_ nem nos escores de asma, na administração de β-2 agonista entre as técnicas inalatórias (nebulizador e MDI-espaçador) em pacientes asmáticos atendidos em emergências pediátricas.

## INTRODUCTION

Asthma is the most common chronic disease in childhood and has been subject of studies for at least two decades, due to its increasing prevalence.^1^ The symptoms are persistent, recurrent, and entirely related to bronchial hyperresponsiveness.^2^ In addition to genetics, some environmental risk factors are implicated in the disease onset: exposure to dust, pets, cockroaches, mold, fungi, viruses, grass, among others.^3^

The prevalence of asthma in a child’s first three years of life may reach 50%. Half of persistent cases begin before the age of three and 80% before the age of six.^4^ The chance of controlling the disease’s morbidity in children as they grow up through treatment is significant.^4^ Dry cough, physical activity-induced respiratory failure, wheezing, chest pain or tightness, temporary respite in respiratory tract, and fatigue are some of the asthma-related morbidities.^5^

In cases of recurrence of acute exacerbations of the disease, current guidelines recommend the use of short-acting bronchodilators (β 2 agonists) to reverse airflow obstruction and treat patients.^6^ At emergency units, bronchodilators are administered by inhalation or nebulization techniques aided by spacers (MDI spacer).^6^ Nebulization has historically been the preferred method for β 2 agonists administration in young patients or patients unable to coordinate inhalation, with the use of inhalation technique aided by MDI spacer, due to lack of understanding of the inhalation technique.^7^ However, in clinical routine and under the supervision of trained professionals, the MDI-spacer technique may be just as effective as nebulization.^8^ Although the efficacy of nebulization is broadly acknowledged, the method has several disadvantages. Studies show that nebulization may be ineffective in delivering aerosolized medicine compared to the combination MDI-spacer.^9^

That being said, the purpose of this study was to compare the efficacy of pediatric asthma treatment by nebulization with MDI spacer in asthmatic children and adolescents assisted at pediatric emergency services.

## METHOD

A research logic was applied to identify the major original, randomized controlled trials that compared the use of nebulization and MDI-spacer techniques in children and adolescents with asthma.

For inclusion in this systematic review, articles had to be randomized controlled trials, with or without the use of placebo. In addition, they should address efficacy comparison between nebulization and MDI-spacer techniques for the treatment of pediatric asthma. Articles without this information were excluded, as were systematic reviews or meta-analyses.

The search strategy was logic based on specific descriptors (in English, Portuguese, and Spanish), linked to the Boolean operator (AND), using parentheses to delimit logic intercalations and quotation marks to identify compound words, as follows: English (nebulizer AND inhaler AND asthma). Searches were made on PubMed, Scientific Electronic Library Online (SciELO), and ScienceDirect databases in October 2016, without restrictions as to period of publication. In order to avoid including an excessive number of articles, searches were delimited in the following fields: heading, keywords, and abstract. Thus, all three descriptors should necessarily appear in at least one of the three search fields (heading, keywords and abstract).

In addition to fields, no limiting filters such as article language or target audience have been added. Articles were exported to MEDLINE and RIS extensions. Data were imported by means of a software intended to the elaboration of systematic reviews (State of the Art through Systematic Review, StArt)^10^, which helped identifying duplicates, as well as excluded and included articles. These analyses were performed separately by three researchers and revised by more than one reviewer.

Articles eligibility criteria were the following, for both inclusion and exclusion:

1. articles selected by the three researchers were automatically included;
2. articles not selected or selected by only one of the researchers were automatically excluded;
3. articles included by two researchers were analyzed by a reviewer and, if they met criteria, they were included.

For the meta-analysis, after articles inclusion and identification of the outcome variables, the software Review Manager (RevMan)^11^ was used, and bivariate differential mean statistics was applied (intergroup estimation - MDI-spacer versus nebulizer), with 95% confidence interval (95%CI), to estimate outcome means.

In the meta-analysis, four outcomes were investigated while comparing the use of metered-dose inhaler to spacer and nebulization: heart rate; respiratory rate; O_2_ saturation; and clinical asthma score, that is, evaluation of respiratory rate, presence of wheezing, cyanosis, chest retractions and transcutaneous oxygen saturation, with scores ranging from 0 to 15 points.^8^

In order to register the systematics, the study was previously registered on the website of the Centre for Reviews and Dissemination (PROSPERO) (http://www.crd.york.ac.uk/PROSPERO), identified by registration number CRD42015023199.

## RESULTS

**Figure 1:**
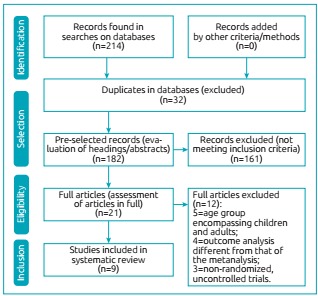
Study design and selection of articles.

In total, 212 articles were retrieved in electronic searches (PubMed=114, ScienceDirect=91; SciELO=7). Initially, 32 articles were excluded for being duplicated and 161 for not meeting inclusion criteria after reading screening of headings and abstracts. Twenty-one articles were selected for full reading and, of these, 12 were excluded after full reading (five of them did not distinguish between pediatric and adult patients, four had different outcome analyses compared to those assessed in the meta-analysis, three were non-randomized or uncontrolled trials), so nine papers were included in our meta-analysis (Figure 1).

Table 1 shows the results of the nine studies included in the systematic review, pointing to similarities between the mean heart and respiratory rates, oxygen saturation and forced expiratory volume in the first second (FEV_1_), after treatment with nebulization and MDI-spacer techniques.

**Table 1:** Characteristics of patients addressed in studies selected

	Nebulizer (n=641)		MDI-spacer (n=666)	
	Mean±SD	n (%)	Mean±SD	n (%)
Males		363 (56.6)		359 (53.9)
Age	6.78±2.61		6.98±2.74	
Heart rate (bpm)	132.39±18.85		129.29±21.12	
Respiratory rate (mpm)	36.80±11.12		36.63±11.05	
O_2_ saturation (%)	95.07±2.07		95.09±2.78	
Asthma clinical score (0-15)	6.30±1.59		6.20±1.26	
VEF_1_ (%)	47.95±9.76		46.75±10.62	

MDI-spacer: metered-dose inhaler-aided spacer; SD: standard deviation; bpm: beats per minute; mpm: movements per minute; O_2_: oxygen; VEF_1_: forced expiratory volume in the first second.

**Figure 2:**
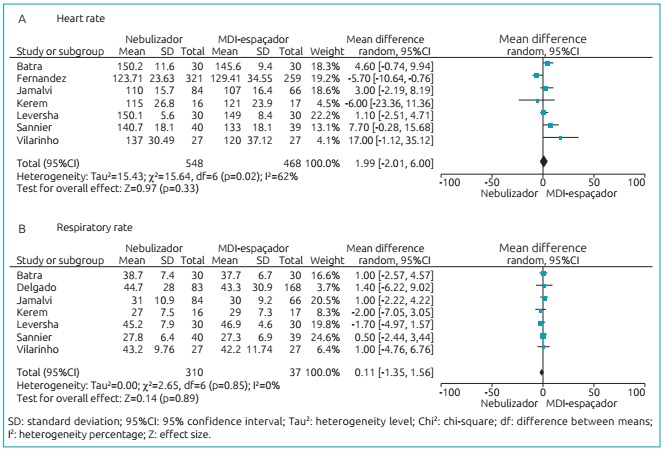
Difference between heart (A) and respiratory (B) rates according to nebulization techniques and the use of MDI-spacer.

**Figure 3:**
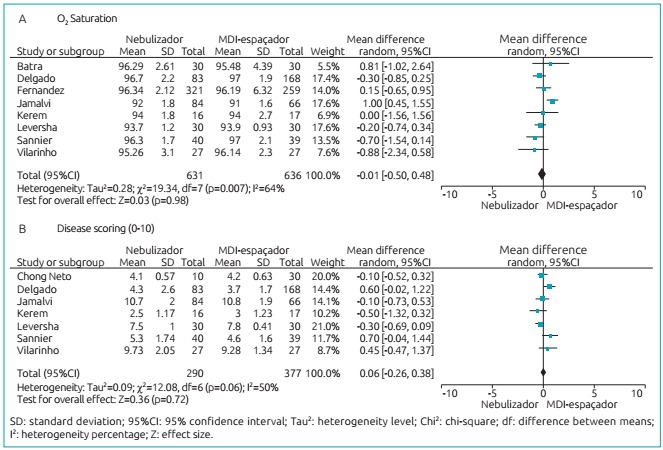
Difference between O2 saturation (A) and asthma score (B) according to nebulization techniques and the use of MDI-spacer.

Chart 1 shows general data of studies and a synthesis of final outcomes, also pointing no differences between the techniques. Figures 2 and 3 show, through meta-analysis, that inhalation can be as effective as the nebulization technique, and no significant differences between have been found.

**Chart 1:** Characteristics of studies evaluated in systematic review, with 1,307 children evaluated in total (641 in nebulizer group and 666 in MDI-spacer group).

Authors	Year	Country	Age (years)	N (subjects)	Nebulizer	MDI-spacer	Outcome
Batra et al*.* ^12^	1997	India	1 a 12	60	0.15 mg/kg salbutamol (max. 5.00 mg)	200 µg salbutamol	MDI-spacer is as effective as aerosol nebulizer (salbutamol) for acute asthma exacerbation in children
Chong-Neto et al*.* ^13^	2005	Brazil	6 a 18	580	5 mg/mL albuterol	400 µg salbutamol	Nebulizer has higher cost and consumes more drugs than MDI-spacer
Delgado et al*.* ^14^	2003	USA	0 a 2	40	0.15 mg/kg salbutamol (max. 5.00 mg)	300 µg salbutamol	Metered-dose inhalers with spacers may be as effective as nebulizers for emergency treatment of wheezing in children aged ≤2 years
Fernandez et al*.* ^15^	2004	Spain	0 a 14	251	2.5 mg/mL salbutamol	200 µg salbutamol	MDI-spacer is as effective as aerosol nebulizers (salbutamol) for acute asthma exacerbation in children
Jamalvi et al*.* ^16^	2006	Pakistan	0 a 15	150	0.3 mg/kg salbutamol (max. 5.0 mg)	200 µg salbutamol	MDI-spacer is an effective alternative, as well as nebulizers, to treat children with exacerbation of acute asthma at emergency rooms
Kerem et al*.* ^17^	1993	Canada	6 a 14	33	5 mg/mL albuterol	400 µg salbutamol	MDI-spacer and nebulizers are equally effective to administer β 2 agonists in children with acute asthma
Leversha et al*.* ^18^	2000	New Zealand	1 a 4	60	2.5 mg/mL salbutamol	600 µg salbutamol	MDI-spacer is a low-cost alternative to administer salbutamol in children with moderate and severe acute asthma
Sannier et al*.* ^19^	2006	France	4 a 15	79	0.15 mg/kg salbutamol (max. 3.00 mg)	300 µg salbutamol	MDI-spacer is a low-cost alternative for the administration of salbutamol to children with acute asthma at emergency rooms
Vilarinho et al*.* ^20^	2003	Brazil	0 a 11	54	250 µg/drop salbutamol	100 µg salbutamol per 3 kg of weight	MDI-spacer can be used to administer salbutamol to children in wheezing crisis, with some advantages over the nebulizer

N: total subjects included in studies; MDI-spacer: metered-dose inhaler with spacer.

The main outcomes of this study are presented in Figures 2 and 3, corroborating no differences between the outcomes evaluated when comparing nebulizer versus MDI-spacer to administer β 2 agonist as to: heart rate (difference - Df: 1.99 [95%CI -2.01-6.00], p=0.33); respiratory rate (Df: 0.11 [95%CI -1.35-1.56], p=0.89); O_2_ saturation (Df: -0.01 [95%CI -0.50-0.48], p=0.98); and asthma clinical score (Df: 0.06 [95%CI -0.26-0.38], p=0.72).

## DISCUSSION

This meta-analysis shows that the administration of bronchodilators (salbutamol) by means of MDI-spacer has the same effects when applied by inhalation technique through nebulization, with the advantage of drug preparation/administration time and cost-effectiveness. In all nine studies analyzed, conclusions are unanimous as to the similarity in responses to treatment of acute asthma recurrences in moderate and severe cases of children assisted at pediatric emergency rooms.

Upon analysis of the four outcomes evaluated (heart rate, respiratory rate, O_2_ saturation, and asthma clinical score), no significant differences were found in *forest plot* (Df: 1.99 [95%CI -2.01-6.00], p=0.33); (Df: 0.11 [95%CI -1.35-1.56], p = 0.89); (Df: -0.01 [95%CI -0.50-0.48], p=0.98), and (Df: 0.06 [95%CI -0.26-0.38], p=0.72), respectively. This validates the conclusions by the study’s authors.

Batra^12^ compared the efficacy of inhalation techniques by both nebulization and MDI-spacer, with salbutamol being administered to 60 children aged 1 to 12 years with acute asthma treated at an emergency room. Heart rate, respiratory rate, paradoxical pulse, analysis of arterial blood gases, and peak expiratory flow rate were observed in addition to inhalation therapy. Subjects were randomly divided into two groups for the administration of salbutamol by nebulizer or MDI-spacer. Response to treatment was assessed sequentially at 20, 40 and 60 minutes after initiation of therapy. Conclusion is that MDI-spacer is as effective as the nebulization technique for the administration of salbutamol in acute asthma exacerbation in children.

Chong Neto et al.^13^ assessed efficacy, adverse events and treatment cost of acute asthma crisis, using inhalation techniques to administer salbutamol via nebulization and MDI-spacer (both industrial and hand-made), in addition to powder inhaler. Evaluations were performed at 0, 20, 40, and 60 minutes after the application of salbutamol and placebo by means of another device. Forty children in acute asthma crisis aging 11±3.5 years were assessed. Clinical and pulmonary function scores were used, and drug and the inhalation device costs were calculated. Both clinical scores and the change in forced expiratory volume in the first second (FEV_1_) were similar in the groups at the end of the study, with higher heart rate variation for the inhalation by nebulization group compared to MDI-spacer (both hand-made and industrial devices) or metered-dose inhalation techniques by dry-powder devices (p=0,004). The nebulizer and the hand-made spacer caused more tremors (p=0.020).

The cost of treatment per patient was higher in the groups using nebulizer and industrial spacer (R$ 22.31 and R$ 16.58, respectively) (p=0.0001). In conclusion, the nebulization technique was more expensive and employed more drugs to achieve the same efficacy. The hand-made spacer was the cheapest tool, but also related to more adverse events than the industrial device and the powder inhaler. The industrial spacer was as expensive as the nebulizer, but safer. The powder inhaler was cheaper and caused fewer tremors, but tachycardia events were similar to the hand-made spacer’s.

Delgado et al.^14^ investigated whether the administration of salbutamol by MDI-spacer is as effective as by nebulization to treat wheezing in children aged 2 years or younger at a pediatric emergency department. A total of 168 children from a convenience sample of wheezing cases participated in the study. The treatment was salbutamol administered every 20 minutes by a single (blind) investigator for group assignment. As primary outcomes, admission rate, pulmonary function, and oxygen saturation were determined at the beginning of treatments and ten minutes later. As a result, the nebulizer group showed better lung function compared to the MDI-spacer group (p=0.002). The analyses also showed lower admission rates in the MDI-spacer group, especially among children with more severe asthma exacerbation, however they came to the conclusion that MDI-spacer can be as effective as nebulizers for emergency treatment of wheezing in children up to 24 months old.

Fernandez et al.^15^ analyzed the efficacy of salbutamol by MDI-spacer compared to the nebulizer to treat acute asthma at a pediatric emergency unit. In total, 580 children up to 14 years of age participated in the sample. No significant differences were found between both groups as to oxygen saturation or heart rate. The number of inhaled bronchodilator doses was also similar (1.42±1.01 versus 1.45±0.98), as well as the number of children requiring observation, admission to hospital or re-referral to medical care. To conclude, the authors reported the same findings as previous studies: administering bronchodilators by MDI-spacer may be an alternative as effective as using nebulizers to treat children with acute asthma exacerbations seen at pediatric emergency rooms.

Jamalvi et al.^16^ sought to determine whether administration of β 2 agonist by MDI-spacer is as effective as by nebulizer for acute asthma. Their study was conducted in the Emergency Room of the National Institute of Child Health (NICH) in Karachi, Pakistan, between October 2000 and March 2001. Participants included 150 children aged 6 months or older and presenting with acute asthma exacerbation. They were categorized into mild, moderate, and severe asthma, and then were randomly assigned to two groups (nebulization and MDI-spacer). Both baseline characteristics and asthma severity were recorded. The variables dyspnea, accessory muscle use, cyanosis, respiratory rate, heart rate, blood pressure, oxygen saturation, paradoxical pulse, pulmonary auscultation, and peak expiratory flow before and after inhalation therapy were also kept track of.

As results, the authors reported that there were no differences between the groups for demographic characteristics or for outcome measures, except for intragroup assessment of peak flow, with a significant increase in both groups (baseline versus post-therapy data); however, values were not statistically significant when compared to each other. In conclusion, they also described the same findings reported by previous studies, that is, the use of MDI-spacer can be an alternative just as effective as nebulization to treat children with acute asthma exacerbation at pediatric emergency rooms.

Kerem et al.^17^ compared the response of children with acute asthma to inhaled salbutamol after its administration by nebulizer or MDI-spacer. Thirty-three children aged 6 to 14 years participated in the study and had FEV_1_, asthma clinical score, heart rate, respiratory rate, and oxygen saturation assessed before and after intervention. As a response, with the exception of heart rate, which increased in the nebulizer group and decreased in the MDI-spacer group (p<0.05), no difference was found in clinical score improvement, respiratory rate, oxygen saturation, or FEV_1_. The authors conclude that MDI-spacer and nebulizer are equally effective means of administering β 2 agonists to children with acute asthma.

Leversha et al.^18^ compared the cost-effectiveness of salbutamol via MDI-spacer versus nebulizer in children with (moderate and severe) acute asthma seen at a pediatric emergency room. Sixty children aged 1 to 4 years participated in the study. Disease scores, heart and respiratory rates, auscultation findings, and oxygen saturation (before and after intervention) were evaluated. Both baseline characteristics and asthma severity were similar in both treatment groups.

MDI-spacer was as effective as the nebulizer for clinical score, respiratory rate and oxygen saturation, but produced more wheezing reduction (p=0.03). In addition, increased heart rate was observed in the nebulizer group (p<0.01). No differences in rates of tremor or hyperactivity were found. The mean cost was lower in the MDI-spacer group (NZ$ 825) compared to the nebulizer group (NZ$ 1,282) (p=0.03). The authors concluded that the MDI-spacer can be a low-cost alternative to nebulizer when treating moderate and severe acute asthma.

Sannier et al.^19^ compared the efficacy of β 2 agonist administration via nebulization or MDI-spacer for moderate and severe asthma recurrence events. A total of 79 children aged 4 to 15 years, treated at an emergency room for moderate and severe asthma, participated in the study. Hospitalization rate, respiratory and heart rates, and peak expiratory flow (PEF), as well as recurrence cases, were assessed. Both groups differed in terms of respiratory distress duration before arriving at the emergency room (p<0.02), with no difference in other variables after interventions. Conclusion was that the efficacy of both techniques is similar and that the use of MDI-spacer should be more frequent in emergency rooms.

Vilarinho et al.^20^ conducted a clinical trial comparing MDI-spacer and the nebulizer for the administration of salbutamol to treat wheezing crisis in children, and a convenience sample of children in moderate wheezing crisis was assessed, the subjects being randomly assigned to two groups according to the inhalation device used for the administration of salbutamol (nebulizer or MDI spacer). The parameters used to compare groups’ outcomes were grouped into score table and consisted of clinical signs commonly used to measure the severity of an asthmatic crisis (level of consciousness, skin color, dyspnea intensity, draw intensity, expiratory time, airflow and wheezing) and transcutaneous oxygen saturation, obtained before treatment and 15 minutes after intervention with salbutamol.

As additional data, time for preparation and use of medications was measured, costs involved in both forms of treatment were computed, and patients’ companions were questioned about their level of satisfaction with the treatments. Fifty-four children aged between 22 days and 11.7 years participated in the study. Groups were not different demographically as to clinical scoring or oximetry values. Comparison of clinical parameters and oxygen saturation between groups did not show significant differences after salbutamol was administered. Time of preparation and administration of the medication, as well as the cost of treatment, were significantly lower in the MDI-spacer group. Family satisfaction levels were similar in both groups. As a conclusion, the authors found that MDI-spacer can be used to administer salbutamol in children with wheezing crisis, with some advantages over the nebulizer.

The main limitation of the studies evaluated was the lack of standardization for pulmonary function evaluation, like in the case of FEV_1_, forced vital capacity (FVC) and Tiffeneau index, which evaluates the relationship between these variables (FEV_1_/FVC), as well as levels of exhaled nitric oxide (FeNo), for these elements are the main markers of asthma control. This was, therefore, the main limitation of our meta-analysis, which was not able to show improvement in lung function markers with the use of both inhalation techniques, although acceptable heterogeneity was stated for the disease in all four outcome variables evaluated, with minimum values for respiratory rate (I^2^=0%) and maximal for O_2_ saturation (I^2^=64%), showing that even with a disease as heterogeneous as asthma, the studies employ acceptable methodological similarities for such outcome variables.

Rescue treatment of asthma exacerbations in pediatric emergency units is usually made with administration of salbutamol via nebulization. This study shows that there are no differences in the administration of β-2 agonist via MDI-spacer or nebulizer.
